# NF-κB signaling as a central integrator of inflammation, stress-induced metabolic dysfunction, and cardiomyocyte fate in sepsis-induced myocardial injury

**DOI:** 10.3389/fimmu.2026.1807015

**Published:** 2026-10-20

**Authors:** Haitang Liao, Yuling Zhou, Xiangwen Weng, Dongsheng Ren, Wenhui Guo, Chenyang Duan, Ming Wang, Zhenchun Luo

**Affiliations:** 1 Department of Critical and Emergency Medicine, Chongqing Hospital of Traditional Chinese Medicine, Chongqing, China; 2 Department of Anesthesiology, The Second Affiliated Hospital of Chongqing Medical University, Chongqing, China; 3 Basic Medicine Research and Innovation Center for Novel Target and Therapeutic Intervention, Ministry of Education, The Second Affiliated Hospital of Chongqing Medical University, Chongqing, China; 4 Department of Cardiology, Chongqing Hospital of Traditional Chinese Medicine, Chongqing, China

**Keywords:** cardiomyocyte fate, inflammatory amplification, metabolic dysfunction, NF-κB signaling, sepsis-induced myocardial injury

## Abstract

Sepsis-induced myocardial injury is a frequent and life-threatening complication of sepsis, characterized by transient yet profound cardiac dysfunction that markedly worsens patient outcomes. Despite extensive investigation, the molecular mechanisms linking systemic inflammation to myocardial metabolic failure and cardiomyocyte loss remain incompletely understood. Emerging evidence indicates that nuclear factor-κB (NF-κB) signaling functions as more than a canonical inflammatory pathway in this context. In this review, we propose a unifying framework in which NF-κB serves as a central integrator linking inflammatory stress, stress-induced metabolic dysfunction, and cardiomyocyte fate in sepsis-induced myocardial injury. We summarize how NF-κB-driven inflammatory amplification, through crosstalk with MAPK signaling and immune cell activation, reshapes the myocardial immune microenvironment. We further discuss how NF-κB interfaces with oxidative stress, mitochondrial dysfunction, redox imbalance, and ferroptosis-related metabolic stress, thereby disrupting cardiomyocyte homeostasis. Importantly, NF-κB is highlighted as a context-dependent determinant of cardiomyocyte fate, modulating apoptosis, pyroptosis, and non-apoptotic cell death programs under septic conditions. Finally, we discuss emerging NF-κB-directed therapies and the need for tissue-specific combination strategies. This integrative framework may guide more precise therapeutic development for sepsis-induced myocardial injury.

## Graphical Abstract

**Figure:**
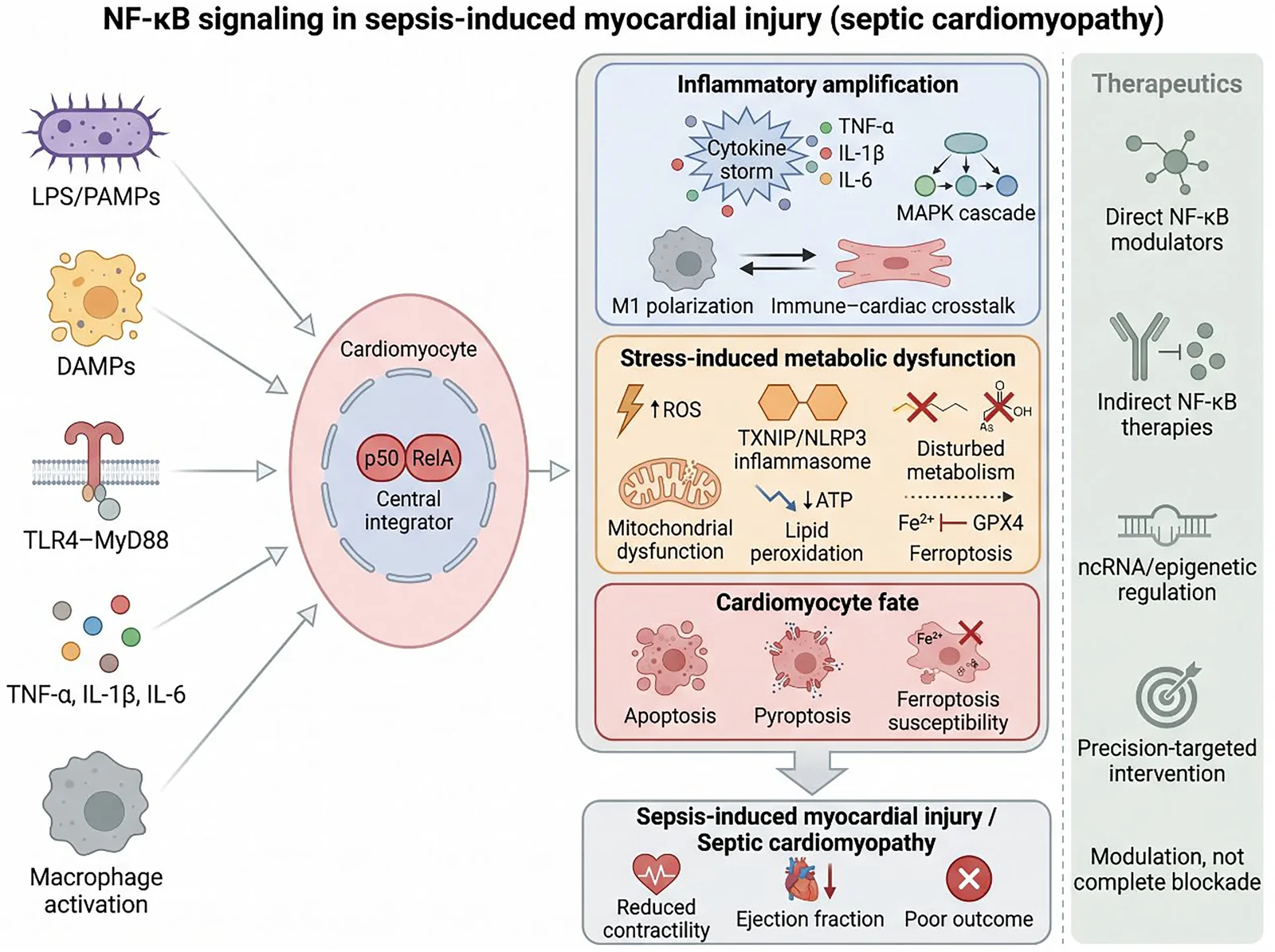

## Highlights

- NF-κB integrates inflammatory signaling, metabolic stress, and cardiomyocyte fate in sepsis-induced myocardial injury.
- Crosstalk between NF-κB, MAPK pathways, and immune cells amplifies myocardial inflammation and metabolic dysfunction.
- Targeting NF-κB-centered networks offers opportunities for precision and combination therapies in septic cardiomyopathy.

## 1 Introduction

Sepsis-induced myocardial injury (SIMI), also referred to as sepsis-induced cardiomyopathy (SIC), is a frequent and clinically significant complication of sepsis, characterized by transient but profound impairment of cardiac structure and function (1–6). Clinical studies have reported that myocardial dysfunction occurs in approximately 40–60% of patients with severe sepsis or septic shock and is strongly associated with increased mortality, prolonged intensive care unit stay, and poor long-term outcomes. Although cardiac dysfunction in sepsis is often described as reversible, its presence markedly worsens short-term prognosis and contributes to multi-organ failure (7–10). Importantly, SIMI is not simply a consequence of reduced coronary perfusion or primary ischemic injury. Accumulating evidence indicates that septic myocardial dysfunction arises from a complex interplay between systemic inflammatory responses and cardiomyocyte-intrinsic stress signaling. Excessive release of inflammatory mediators, dysregulated immune cell activation, oxidative stress, and mitochondrial damage collectively disrupt excitation–contraction coupling, calcium handling, and energy metabolism in cardiomyocytes (11, 12). As a result, cardiac depression in sepsis reflects a state of maladaptive stress integration, rather than isolated myocardial suppression. Despite advances in supportive care, there are currently no specific therapies that directly target the molecular mechanisms underlying SIMI. This highlights an urgent need to better understand the signaling networks that coordinate inflammation, metabolic stress, and cardiomyocyte fate decisions during sepsis.

Over the past two decades, extensive experimental studies have identified multiple signaling pathways implicated in septic myocardial injury. These include Toll-like receptor 4 (TLR4)-mediated innate immune activation, mitogen-activated protein kinase (MAPK) cascades, nucleotide-binding oligomerization domain-like receptor family pyrin domain-containing 3 (NLRP3) inflammasome signaling, reactive oxygen species (ROS)–driven oxidative stress, and various forms of regulated cell death such as apoptosis, pyroptosis, and ferroptosis (13). While each of these pathways has been shown to contribute to myocardial injury in sepsis, they are frequently investigated in isolation. Such reductionist approaches, although mechanistically informative, fail to fully explain the dynamic and systemic nature of septic myocardial dysfunction. In reality, inflammatory signaling, metabolic stress, and cardiomyocyte death programs are highly interconnected and mutually reinforcing. A central unanswered question therefore remains: how are these diverse stress signals sensed, integrated, and translated into coordinated cellular responses within the septic heart? Emerging evidence suggests that nuclear factor-κB (NF-κB) signaling occupies a pivotal position within this network. Beyond its classical role as a transcriptional regulator of pro-inflammatory cytokines, NF-κB integrates upstream immune sensing signals with downstream metabolic stress responses and cell fate decisions (14). Through extensive crosstalk with MAPK pathways, inflammasome priming, oxidative stress signaling, and mitochondrial regulation, NF-κB functions as more than a linear inflammatory pathway—it acts as a central integrator that orchestrates the myocardial response to septic stress.

In this review, we propose a unifying framework in which NF-κB acts as a central integrator of systemic inflammation, stress-induced metabolic dysfunction, and cardiomyocyte fate in sepsis-induced myocardial injury. We focus on three interconnected dimensions: inflammatory amplification through innate immune and cytokine signaling; metabolic dysfunction involving oxidative stress, mitochondrial impairment, and redox imbalance; and cardiomyocyte fate, including apoptosis, pyroptosis, and ferroptosis. Rather than describing these pathways in isolation, we emphasize their crosstalk within NF-κB-centered regulatory networks. We also compare findings across experimental models, including LPS-induced injury, cecal ligation and puncture, isolated cardiomyocytes, and immune cell-based systems, to highlight differences in the timing, cellular source, and downstream consequences of NF-κB activation. Particular attention is given to model dependence, cell-type specificity, temporal dynamics, and translational limitations. Beyond established TLR4/MyD88 and MAPK pathways, we further discuss emerging regulatory layers, including non-coding RNAs and epigenetic modulators, and their therapeutic implications.

## 2 Canonical and non-canonical NF-κB signaling in sepsis

### 2.1 Overview of NF-κB signaling architecture

The nuclear factor-κB (NF-κB) family comprises a group of transcription factors that play central roles in immune regulation, stress responses, and cell fate determination. In mammalian cells, NF-κB signaling is classically divided into canonical and non-canonical pathways, which differ in their upstream activators, molecular components, and biological outputs. The canonical NF-κB pathway is the predominant and most rapidly activated signaling cascade in sepsis. It is typically initiated by pattern recognition receptors, particularly Toll-like receptor 4 (TLR4), following recognition of pathogen-associated molecular patterns such as lipopolysaccharide (LPS) (15–17). Upon activation, TLR4 recruits adaptor proteins including myeloid differentiation primary response 88 (MyD88), leading to the assembly of downstream signaling complexes and activation of the IκB kinase (IKK) complex (18–20). Phosphorylation of inhibitor of κB (IκB) proteins by IKK triggers their proteasomal degradation, allowing the NF-κB heterodimer—most commonly p65 (RelA)/p50—to translocate into the nucleus and initiate transcription of target genes. These genes include pro-inflammatory cytokines, chemokines, adhesion molecules, and stress-responsive mediators that are highly relevant to septic myocardial injury. In contrast, the non-canonical NF-κB pathway is activated by a more restricted set of stimuli, such as certain members of the tumor necrosis factor receptor superfamily, and involves NF-κB–inducing kinase (NIK)-dependent processing of p100 to p52, resulting in RelB/p52 nuclear translocation. Although the non-canonical pathway contributes to immune cell differentiation and chronic inflammatory states, its role in acute sepsis-induced myocardial injury appears less dominant (21, 22). Nevertheless, emerging evidence suggests that non-canonical signaling may modulate immune cell phenotypes and inflammatory persistence in prolonged or unresolved sepsis, warranting further investigation.

### 2.2 Activation of NF-κB in sepsis and myocardial tissue

During sepsis, NF-κB signaling is robustly activated by a broad spectrum of upstream triggers. Circulating microbial products such as LPS, as well as endogenous damage-associated molecular patterns (DAMPs) released from injured tissues, converge on TLRs and other innate immune sensors to initiate NF-κB activation (23–27). In parallel, elevated levels of inflammatory cytokines—including tumor necrosis factor-α, interleukins, and other mediators—further amplify NF-κB signaling through autocrine and paracrine mechanisms (28–30). Importantly, NF-κB activation in sepsis is not confined to immune organs but occurs directly within the myocardium. Both cardiomyocytes and resident or infiltrating immune cells, particularly macrophages, exhibit pronounced NF-κB activation in response to septic stimuli. In cardiomyocytes, NF-κB signaling is rapidly induced by inflammatory mediators and oxidative stress, leading to transcriptional reprogramming that affects contractile function, mitochondrial integrity, and stress adaptation (31–33). In immune cells, especially macrophages, NF-κB activation governs inflammatory polarization, cytokine production, and the propagation of myocardial inflammation through immune–cardiac crosstalk (34–36). Crucially, in the context of sepsis, NF-κB activation should not be viewed solely as a trigger for inflammatory gene expression. Instead, accumulating evidence indicates that NF-κB signaling initiates a broader program of functional remodeling, influencing metabolic stress responses, redox homeostasis, mitochondrial function, and regulated cell death pathways. This expanded functional scope positions NF-κB as a central signaling node that translates septic stress into coordinated alterations in myocardial inflammation, metabolism, and cell fate—thereby setting the stage for the complex pathophysiology of sepsis-induced myocardial injury.

## 3 NF-κB as the central driver of inflammatory amplification

### 3.1 TLR4–MyD88–NF-κB axis in septic myocardial inflammation

The Toll-like receptor 4 (TLR4)–MyD88–NF-κB signaling axis represents the principal inflammatory sensing and signal transduction pathway in sepsis-induced myocardial injury (37). As a pattern recognition receptor, TLR4 functions as the primary sensor of both pathogen-associated molecular patterns, such as lipopolysaccharide, and endogenous danger-associated molecular patterns released from damaged tissues during sepsis. Engagement of TLR4 rapidly converts systemic infectious stress into intracellular inflammatory signaling, thereby linking circulating inflammatory cues to myocardial dysfunction. Upon activation, TLR4 recruits adaptor proteins to its intracellular Toll/IL-1 receptor domain, among which myeloid differentiation primary response 88 (MyD88) serves as a critical signal amplification hub (38–41). MyD88-dependent signaling promotes the sequential activation of downstream kinases and the assembly of multiprotein signaling complexes, ultimately leading to robust activation of the IκB kinase (IKK) complex (42–44). Phosphorylation and proteasomal degradation of inhibitor of κB (IκB) proteins release NF-κB dimers—predominantly p65/p50—allowing their rapid translocation into the nucleus.

Once activated, NF-κB acts as the transcriptional executor of septic inflammation, driving the expression of a broad repertoire of pro-inflammatory genes, including cytokines, chemokines, adhesion molecules, and stress-responsive mediators. In the myocardium, this transcriptional program extends beyond immune activation and directly impairs cardiomyocyte function. NF-κB–dependent inflammatory signaling disrupts calcium handling, alters the expression and post-translational modification of contractile proteins, and compromises mitochondrial homeostasis, collectively contributing to reduced myocardial contractility (45–48). Importantly, the TLR4–MyD88–NF-κB axis operates in both cardiomyocytes and cardiac-resident or infiltrating immune cells, creating a feed-forward inflammatory circuit within the myocardial microenvironment (49). NF-κB-driven cytokine release from immune cells amplifies paracrine stress on cardiomyocytes, while cardiomyocyte-intrinsic NF-κB activation further reinforces local inflammatory signaling. Through this bidirectional immune–cardiac crosstalk, the TLR4–MyD88–NF-κB axis establishes a sustained inflammatory milieu that underlies septic myocardial dysfunction (**Figure 1**).

**Figure 1:**
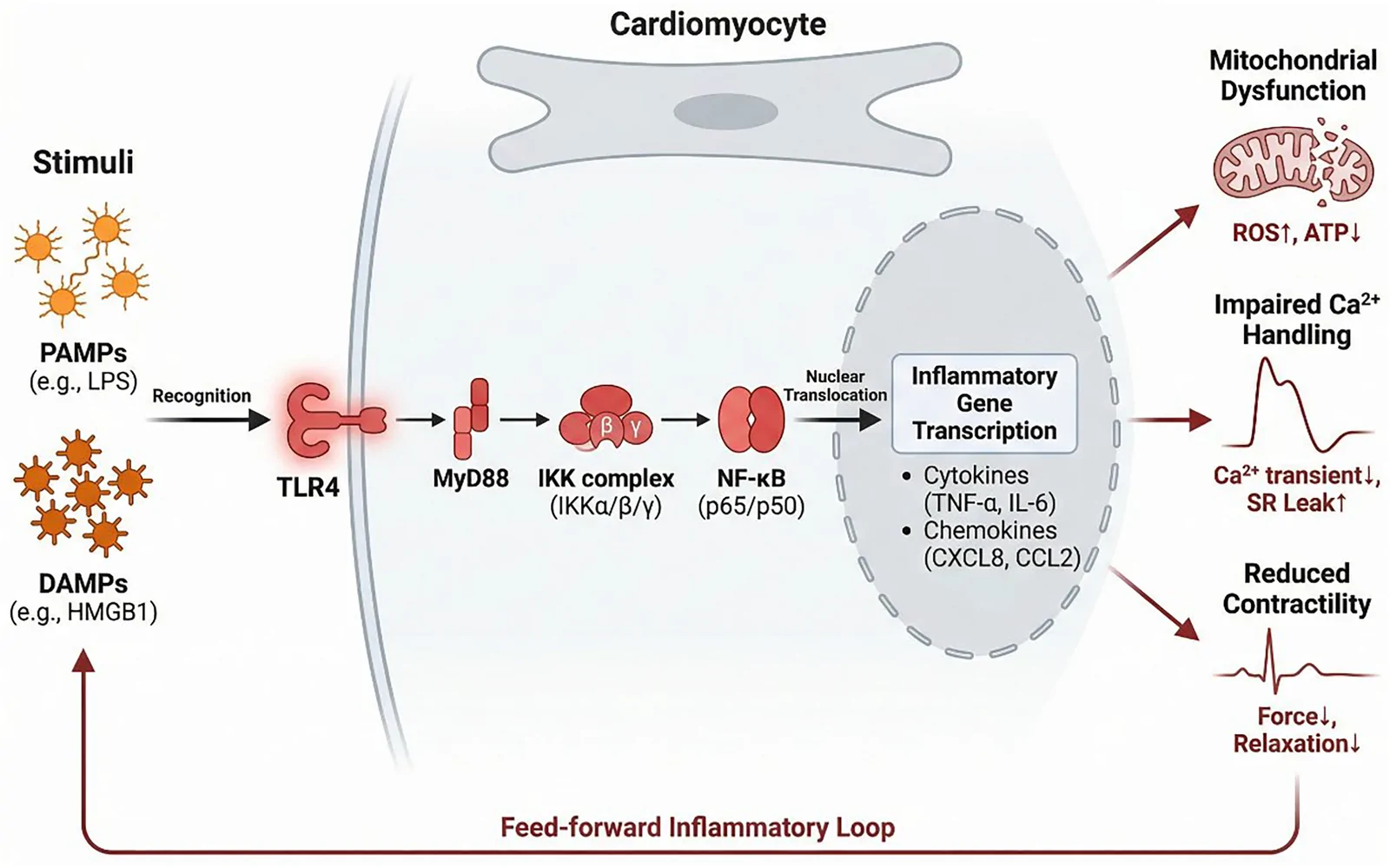
TLR4–MyD88–NF-κB signaling in septic myocardial inflammation. Septic pathogen- and damage-associated molecular patterns activate TLR4 on cardiomyocytes and immune cells, triggering MyD88-dependent NF-κB signaling. Nuclear NF-κB drives inflammatory gene transcription, disrupts calcium handling, contractile protein function, and mitochondrial homeostasis, leading to myocardial dysfunction. Bidirectional immune–cardiac crosstalk establishes a feed-forward inflammatory circuit that sustains septic myocardial injury.

Collectively, these mechanisms position the TLR4–MyD88–NF-κB signaling axis as a central conduit through which innate immune sensing is translated into inflammatory myocardial injury in sepsis, setting the stage for downstream metabolic dysregulation and cardiomyocyte fate decisions discussed in subsequent sections.

### 3.2 Crosstalk between NF-κB and MAPK signaling

In addition to canonical NF-κB activation, sepsis robustly engages mitogen-activated protein kinase (MAPK) pathways, including c-Jun N-terminal kinase (JNK), p38 MAPK, and extracellular signal–regulated kinase (ERK) (50). Rather than functioning as parallel or redundant signaling cascades, MAPK pathways exhibit extensive and dynamic crosstalk with NF-κB signaling, collectively shaping the magnitude, duration, and qualitative features of inflammatory responses in the septic myocardium. MAPK signaling contributes to inflammatory amplification at multiple regulatory levels. Activation of stress-responsive kinases such as JNK and p38 enhances the transcriptional activity of NF-κB by modulating transcriptional coactivator recruitment, chromatin accessibility, and post-translational modification of NF-κB subunits. In parallel, ERK signaling influences the stability, translation efficiency, and temporal persistence of inflammatory mRNAs, thereby extending the functional impact of NF-κB–dependent transcription beyond initial nuclear activation (49). Conversely, NF-κB signaling feeds back to regulate MAPK pathway dynamics. NF-κB-driven transcription controls the expression of upstream MAPK components, scaffold proteins, and dual-specificity phosphatases that fine-tune MAPK activation kinetics. Through these feed-forward and feedback loops, NF-κB and MAPK pathways form an integrated signaling module capable of sustaining inflammatory signaling even after the initial septic stimulus has waned. This cooperative interaction between NF-κB and MAPK pathways is particularly consequential in the myocardium. Cardiomyocytes are highly sensitive to prolonged inflammatory and stress kinase activation, and sustained NF-κB–MAPK crosstalk disrupts calcium handling, impairs mitochondrial function, and promotes maladaptive stress responses (51, 52). In addition, persistent activation of JNK and p38 signaling increases cardiomyocyte susceptibility to regulated cell death, thereby linking inflammatory amplification to downstream myocardial injury (50). Therefore, MAPK signaling should not be viewed as “parallel noise” accompanying NF-κB activation in sepsis. Instead, MAPK pathways function as integral components of an NF-κB-centered inflammatory network that governs not only the intensity but also the persistence and pathological consequences of myocardial inflammation. This integrated signaling architecture provides a mechanistic bridge between inflammatory amplification and the metabolic dysfunction and cardiomyocyte fate decisions discussed in subsequent sections (**Figure 2**).

**Figure 2:**
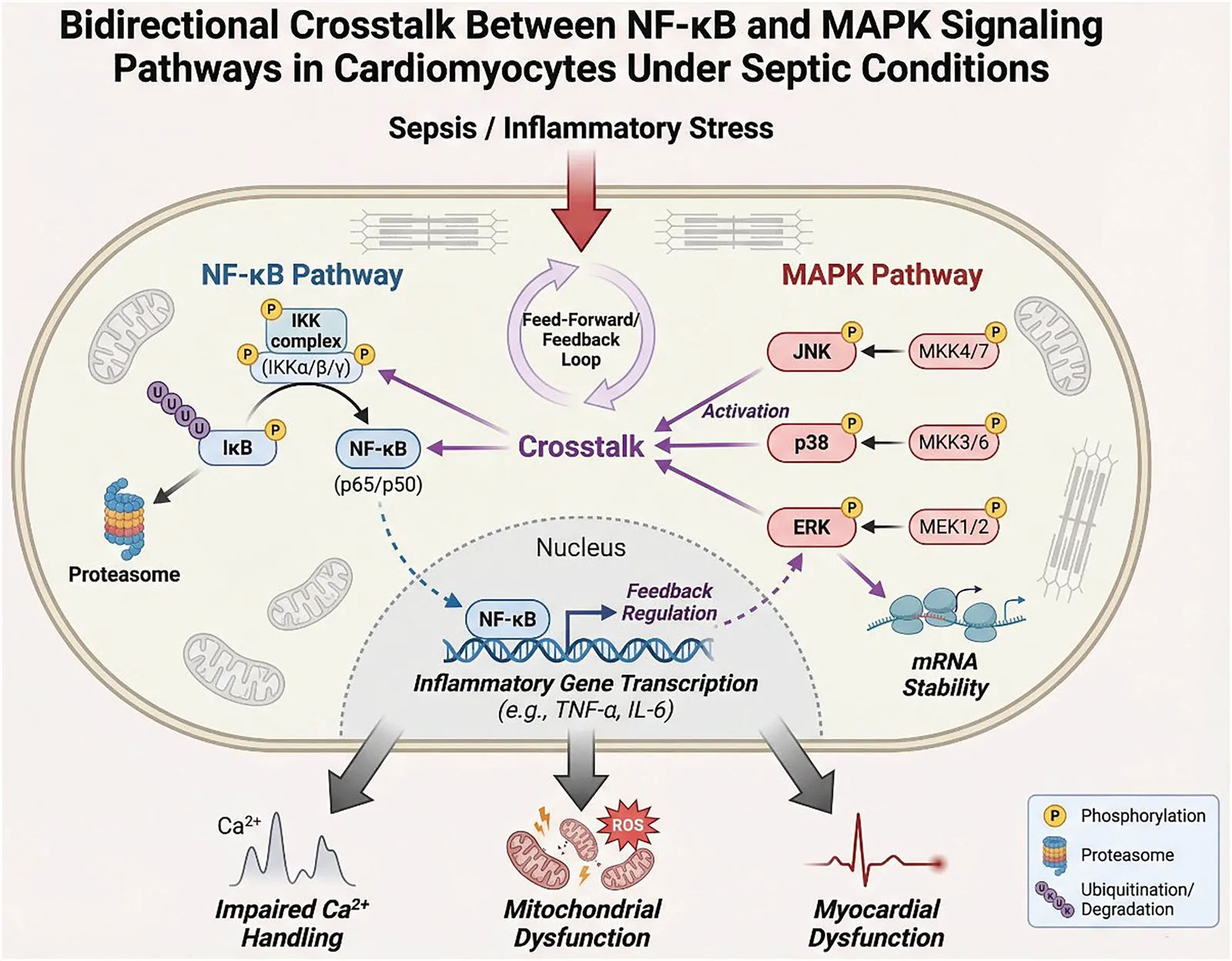
NF-κB–MAPK signaling crosstalk in septic myocardial inflammation. Septic inflammatory stress activates NF-κB and MAPK pathways in cardiomyocytes. JNK and p38 enhance NF-κB transcriptional activity, while ERK prolongs inflammatory mRNA stability. NF-κB feeds back to regulate MAPK signaling, forming feed-forward and feedback loops that sustain inflammation and drive myocardial dysfunction.

### 3.3 NF-κB-mediated immune cell activation and macrophage polarization

Beyond its direct effects on cardiomyocytes, NF-κB plays a pivotal role in shaping the myocardial immune microenvironment during sepsis. Among immune cells, macrophages represent a dominant population infiltrating or residing within the septic heart and serve as key effectors of inflammatory injury (53–56). NF-κB signaling is a central determinant of macrophage activation and polarization. Sustained NF-κB activation promotes a pro-inflammatory, M1-like macrophage phenotype characterized by high production of cytokines, chemokines, and reactive oxygen species (**Figure 3**) (57–60). These activated macrophages amplify myocardial inflammation through paracrine signaling, exacerbate oxidative stress, and impair cardiomyocyte contractility. Conversely, attenuation of NF-κB signaling favors a shift toward anti-inflammatory or reparative macrophage phenotypes, which may contribute to resolution of inflammation and tissue recovery.

**Figure 3:**
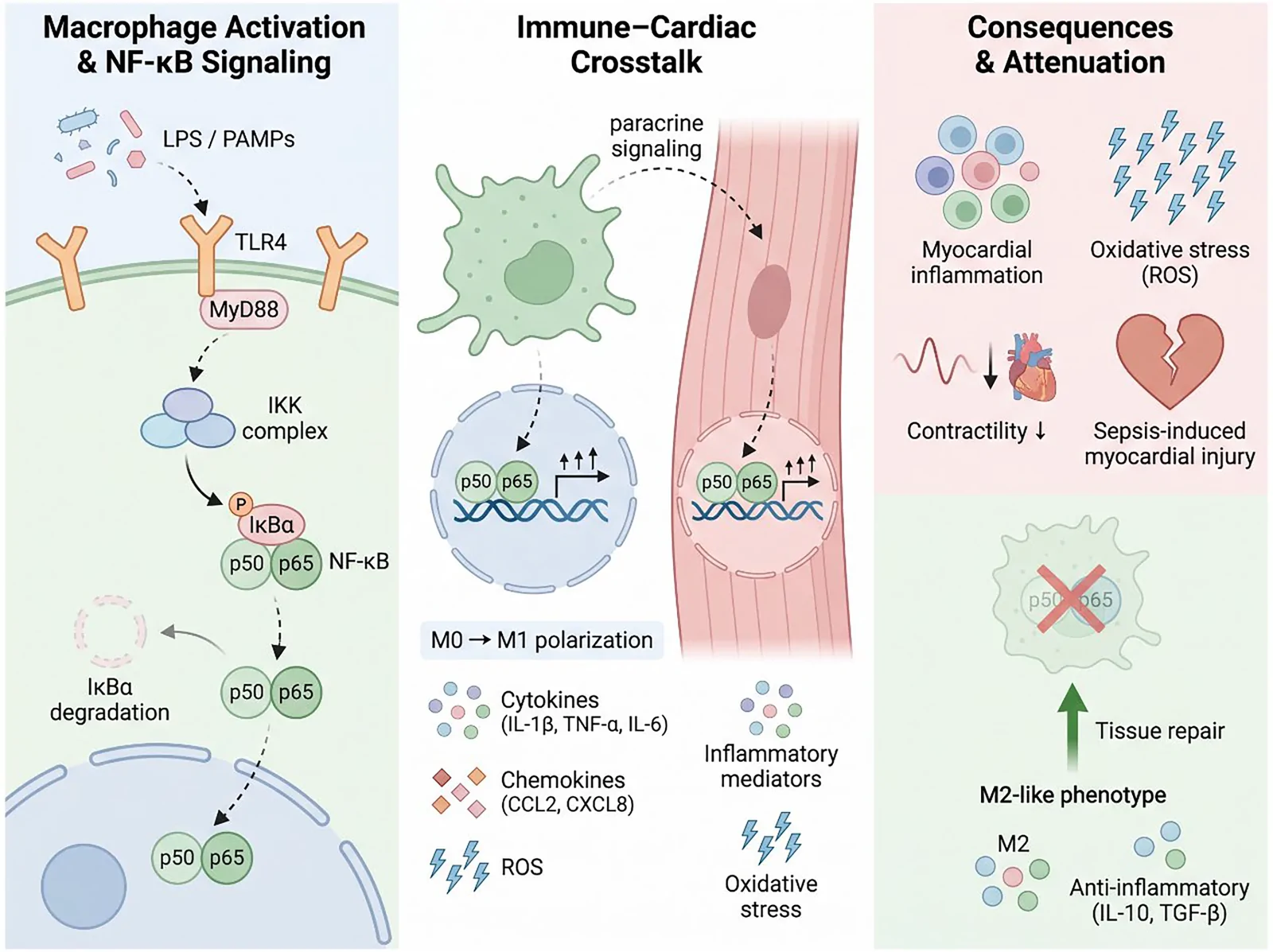
NF-κB–dependent macrophage polarization in septic myocardium. During sepsis, NF-κB activation drives macrophages toward a pro-inflammatory M1-like phenotype, characterized by increased cytokine and ROS production. These activated macrophages amplify myocardial inflammation through paracrine signaling and impair cardiomyocyte function. Attenuation of NF-κB favors a shift toward reparative macrophage phenotypes, contributing to inflammation resolution and tissue recovery.

Importantly, these observations highlight that inflammation in sepsis-induced myocardial injury is not solely a cardiomyocyte-intrinsic event. Instead, it represents a dynamic immune–cardiac interaction, in which NF-κB-centered signaling coordinates immune cell behavior and cardiomyocyte responses within the myocardial microenvironment. This immune-centric perspective provides a crucial conceptual bridge linking inflammatory amplification to downstream metabolic dysfunction and cardiomyocyte fate decisions, as discussed in subsequent sections. The major NF-κB-centered inflammatory signaling modules involved in sepsis-induced myocardial injury are summarized in **Table 1**. Although these studies collectively support the importance of NF-κB-centered inflammatory amplification in sepsis-induced myocardial injury, their findings should be interpreted in a model-dependent manner. LPS-based models often highlight rapid TLR4/MyD88-dependent NF-κB activation in cardiomyocytes and immune cells, whereas polymicrobial sepsis models such as cecal ligation and puncture more closely reflect sustained systemic inflammation, immune cell infiltration, and prolonged myocardial immune remodeling. Similarly, *in vitro* cardiomyocyte studies are useful for defining cell-intrinsic NF-κB responses, but they cannot fully recapitulate macrophage–cardiomyocyte, endothelial–immune, and systemic inflammatory interactions observed *in vivo*. These differences may explain why some studies emphasize direct cardiomyocyte injury, whereas others highlight immune-cell-driven paracrine inflammation or macrophage polarization. Therefore, a key knowledge gap is how NF-κB activation differs across cardiac cell types and disease stages during sepsis. Future studies using cell-specific NF-κB modulation, lineage tracing, and single-cell or spatial transcriptomic approaches are needed to distinguish cardiomyocyte-intrinsic NF-κB signaling from immune-cell-mediated inflammatory crosstalk.

**Table 1:** NF-κB-centered inflammatory signaling modules in sepsis-induced myocardial injury.

Signaling module	Upstream triggers	NF-κB-related mechanisms	Downstream effects in myocardium
TLR4–MyD88 axis	LPS, DAMPs	IKK activation, p65/p50 nuclear translocation	Cytokine release, myocardial inflammation
NF-κB–MAPK crosstalk	TLR4, cytokines	JNK/p38 enhance NF-κB transcriptional activity; ERK stabilizes inflammatory mRNAs	Sustained inflammation, stress kinase activation
Immune cell activation	Circulating pathogens, cytokines	NF-κB-dependent macrophage polarization (M1-like)	Immune–cardiac crosstalk, inflammatory amplification
Cytokine signaling loops	TNF-α, IL-1β	Autocrine/paracrine NF-κB reinforcement	Persistent myocardial inflammatory stress

## 4 NF-κB links inflammatory stress to metabolic dysfunction

### 4.1 Concept of stress-induced metabolic dysfunction in sepsis

In sepsis-induced myocardial injury, stress-induced metabolic dysfunction refers to a maladaptive disturbance of cardiac energy metabolism caused by inflammatory and oxidative stress, rather than a coordinated metabolic adaptation that supports growth, survival, or increased workload (61–63). Under physiological conditions, the adult heart relies predominantly on mitochondrial oxidative phosphorylation, with fatty acid oxidation providing the major proportion of ATP and glucose, lactate, and other substrates contributing flexibly according to energetic demand (64, 65). In the septic heart, this metabolic flexibility is disrupted. Established features include impaired mitochondrial oxidative phosphorylation, reduced ATP-generating capacity, excessive mitochondrial ROS production, abnormal redox balance, altered fatty acid oxidation, disturbed glucose oxidation, and dysregulated mitochondrial biogenesis and quality control.

Importantly, metabolic dysfunction in sepsis-induced myocardial injury should not be regarded as equivalent to the Warburg-type metabolic reprogramming observed in cancer cells or activated immune cells (61, 66). In cancer and immune activation, increased aerobic glycolysis can represent an adaptive program that supports biosynthesis, proliferation, or effector function (67, 68). By contrast, in septic cardiomyocytes, increased glycolytic activity, lactate accumulation, or reduced oxidative metabolism often reflects an inability to maintain efficient mitochondrial ATP production rather than a stable anabolic program. Similarly, unlike exercised skeletal or cardiac muscle, in which mitochondrial biogenesis and substrate switching are coordinated to match increased workload, septic myocardial metabolism is characterized by impaired substrate oxidation, mitochondrial injury, and energetic inefficiency.

The direction of substrate utilization changes in the septic heart remains controversial and appears to depend on experimental model, disease stage, and species. In LPS-based models, inflammatory signaling has been shown to repress PGC-1–dependent metabolic programs and suppress mitochondrial fatty acid oxidation, contributing to lipid accumulation and impaired cardiac energetics (69). However, in early polymicrobial sepsis models, cardiac fatty acid oxidation may be transiently increased, and PPARα-dependent fatty acid oxidation can support early cardiac performance. These apparently divergent findings indicate that cardiac metabolism in sepsis is not a fixed switch from fatty acid oxidation to glycolysis, but a dynamic and stage-dependent loss of metabolic flexibility. Therefore, the term “stress-induced metabolic dysfunction” in this review refers to a pathological state defined by impaired energetic efficiency, unstable substrate utilization, mitochondrial damage, redox imbalance, and failure of adaptive metabolic compensation, rather than by a single uniform metabolic pathway (**Table 2**).

**Table 2:** Defining metabolic features of sepsis-induced myocardial injury compared with adaptive metabolic reprogramming.

Metabolic feature	Sepsis-induced myocardial injury	Cancer/immune-cell metabolic reprogramming	Exercise-induced metabolic adaptation
Overall metabolic logic	Maladaptive loss of metabolic flexibility under inflammatory and oxidative stress	Adaptive support for biosynthesis, proliferation, or effector function	Adaptive matching of substrate use and mitochondrial capacity to workload
Fatty acid oxidation	Variable; suppressed in some LPS models but transiently increased or required in early polymicrobial sepsis	Often reduced or context-dependent depending on cell type	Generally enhanced oxidative capacity with training adaptation
Glucose metabolism	May show increased glycolytic flux or lactate accumulation, but often reflects inefficient mitochondrial oxidation rather than stable Warburg-like adaptation	Aerobic glycolysis supports biosynthesis and immune effector function	Increased glucose uptake can occur with coordinated mitochondrial oxidation
Mitochondrial oxidative phosphorylation	Impaired respiratory efficiency, reduced ATP production, and mitochondrial injury	Variable; may be reduced in Warburg-like states or maintained in oxidative tumors	Enhanced mitochondrial capacity and biogenesis with training
Redox balance	Excessive mitochondrial ROS and oxidative stress	ROS may function as signaling intermediates but can be buffered by adaptive antioxidant systems	Controlled ROS signaling contributes to adaptation
Mitochondrial biogenesis/quality control	Dysregulated PGC-1 signaling, impaired mitochondrial renewal, defective mitophagy or mitochondrial damage accumulation	Context-dependent and often linked to growth/survival programs	Coordinated mitochondrial biogenesis and turnover
Functional outcome	Energy deficit, contractile dysfunction, calcium-handling abnormalities, and susceptibility to regulated cell death	Proliferation, survival, immune activation, or immune exhaustion depending on context	Improved energetic efficiency and stress tolerance

### 4.2 NF-κB and oxidative stress signaling

Oxidative stress represents one of the earliest and most prominent metabolic disturbances in sepsis-induced myocardial injury. Excessive generation of reactive oxygen species (ROS), particularly through NADPH oxidase 2 (NOX2), is a hallmark of septic inflammation and contributes directly to cardiomyocyte dysfunction. In the septic heart, ROS accumulation disrupts redox homeostasis, damages cellular macromolecules, and interferes with excitation–contraction coupling, thereby impairing myocardial performance even in the absence of overt structural injury (70).

NF-κB signaling is intricately intertwined with oxidative stress pathways through a bidirectional and self-amplifying regulatory loop. On the one hand, elevated ROS levels act as potent upstream activators of NF-κB by enhancing IκB kinase (IKK) activity and promoting nuclear translocation of NF-κB subunits (70–73). On the other hand, NF-κB transcriptionally regulates multiple components of the oxidative stress machinery, including enzymes involved in ROS production and inflammatory mediators that further exacerbate redox imbalance. Through this reciprocal interaction, NF-κB not only senses oxidative stress but also actively propagates it, establishing a feed-forward cycle that sustains inflammatory and metabolic injury in septic cardiomyocytes. Thioredoxin-interacting protein (TXNIP) has emerged as a critical molecular nexus linking NF-κB signaling, oxidative stress, and inflammatory injury. TXNIP functions as a redox-sensitive regulator that modulates cellular antioxidant capacity while simultaneously connecting oxidative stress to inflammasome activation. NF-κB-dependent upregulation of TXNIP under septic conditions amplifies oxidative damage and promotes inflammatory signaling, thereby coupling metabolic stress to immune activation (74–77). This convergence further intensifies myocardial injury and accelerates the transition from adaptive stress responses to maladaptive inflammation. Counterbalancing these pro-oxidant and pro-inflammatory pathways, the nuclear factor erythroid 2–related factor 2 (NRF2)/heme oxygenase-1 (HO-1) axis constitutes a major endogenous antioxidant defense system in cardiomyocytes. NRF2 activation induces the expression of cytoprotective and antioxidant genes, preserving redox balance and mitochondrial integrity (78). However, accumulating evidence suggests that NF-κB and NRF2 signaling engage in complex reciprocal regulation. Sustained or excessive NF-κB activation during sepsis can suppress NRF2-driven antioxidant responses, tipping the balance toward oxidative injury (78–80). Disruption of this regulatory equilibrium shifts cardiomyocytes into a pro-oxidant, energy-deficient state that predisposes to mitochondrial dysfunction, impaired contractility, and heightened vulnerability to regulated cell death (**Figure 4**). Oxidative and inflammatory stress may also contribute to the loss of myocardial substrate flexibility by disrupting key control points in glucose and fatty-acid oxidation. In septic myocardium, increased pyruvate dehydrogenase kinase 4 (PDK4) and reduced pyruvate dehydrogenase (PDH) activity restrict the conversion of pyruvate to acetyl-CoA, thereby limiting glucose-derived carbon entry into the tricarboxylic acid cycle (81–83). ROS can further impair PDH activity through oxidative modification, providing an additional link between redox imbalance and defective glucose oxidation (84, 85). In parallel, inflammatory and oxidative stress suppress fatty-acid oxidation by reducing PPARα/PGC-1α-dependent metabolic programs and impairing carnitine palmitoyltransferase-1 (CPT-1), the rate-controlling step for mitochondrial long-chain fatty-acid entry (86–89). These changes help explain why septic cardiomyocytes progressively lose the ability to switch efficiently between glucose and fatty acids, resulting in energetic inefficiency rather than a uniform metabolic shift toward a single substrate.

**Figure 4:**
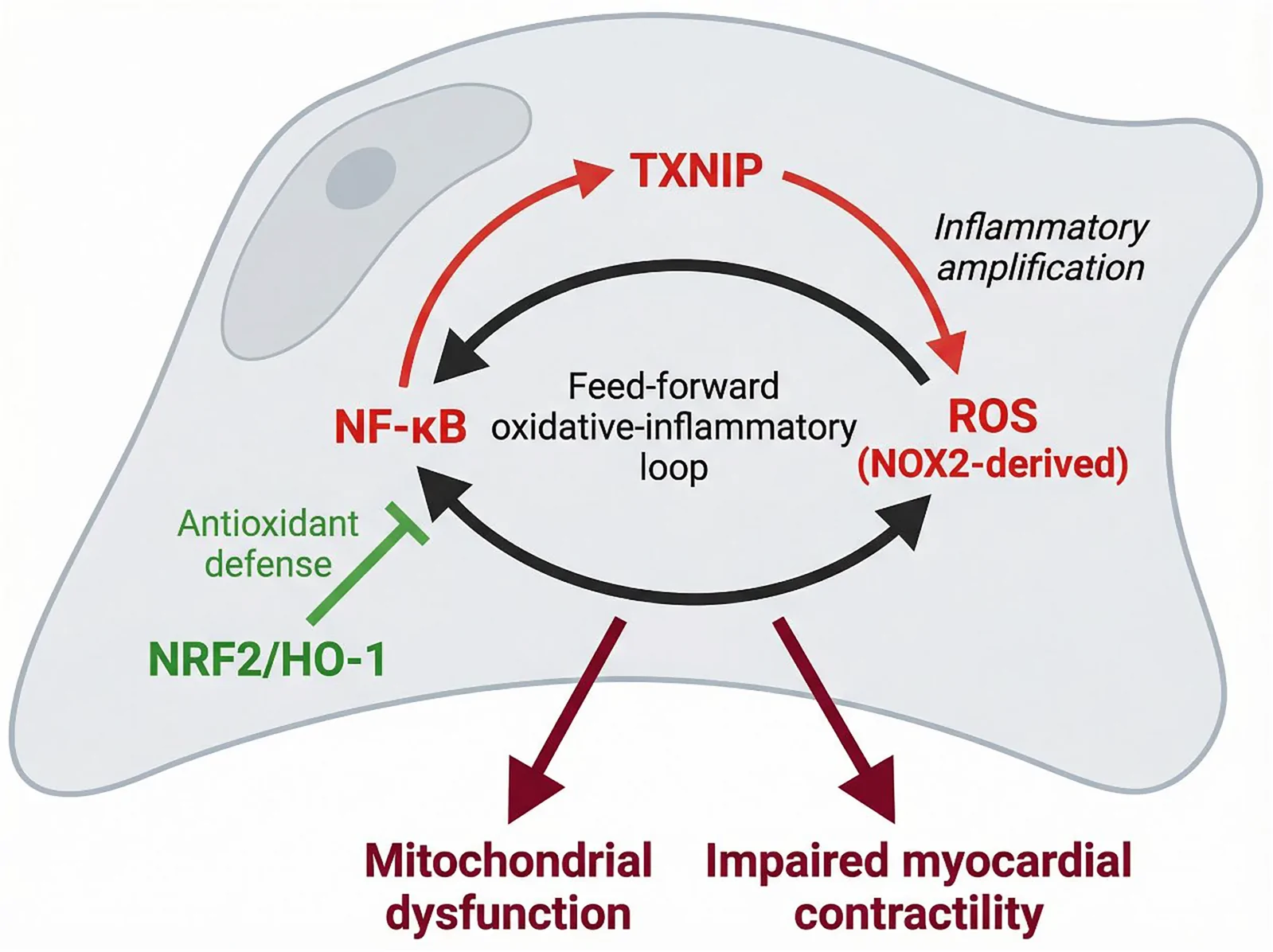
NF-κB–ROS feedback signaling in septic myocardial injury. In septic cardiomyocytes, excessive ROS activates NF-κB signaling, which in turn amplifies oxidative stress through TXNIP-dependent mechanisms, forming a feed-forward inflammatory loop. Suppression of NRF2/HO-1 antioxidant defenses further exacerbates redox imbalance, leading to mitochondrial dysfunction and impaired myocardial contractility.

Collectively, these findings position oxidative stress as a key interface through which NF-κB links inflammatory signaling to metabolic dysfunction in the septic myocardium. By simultaneously responding to and amplifying ROS-driven stress, NF-κB converts transient inflammatory stimuli into persistent redox imbalance, thereby setting the stage for downstream mitochondrial failure and cardiomyocyte fate decisions discussed in subsequent sections. The integrative role of NF-κB in linking inflammatory stress, metabolic dysfunction, and cardiomyocyte fate in sepsis-induced myocardial injury is summarized in **Table 3**.

**Table 3:** NF-κB-mediated integration of inflammatory stress, metabolic dysfunction, and cardiomyocyte fate.

Pathological process	NF-κB-associated pathways	Key metabolic consequences	Functional outcome
Oxidative stress	NOX2/ROS, TXNIP	Redox imbalance, antioxidant suppression	Contractile dysfunction
Mitochondrial impairment	NF-κB-dependent inflammatory mediators	Reduced oxidative phosphorylation, ATP depletion	Reversible cardiac depression
Apoptosis	Cytokine signaling, caspase-3 activation	Energy failure, calcium dysregulation	Cardiomyocyte loss
Pyroptosis	NF-κB-primed NLRP3 inflammasome	Inflammatory cell death	Amplified myocardial inflammation
Ferroptosis	Lipid peroxidation, iron dysregulation	Metabolic stress–driven cell death	Progressive myocardial injury

### 4.3 NF-κB-mediated mitochondrial dysfunction

Mitochondrial dysfunction is a central determinant of cardiac depression in sepsis and represents a critical intersection between inflammatory signaling and metabolic failure (90–95). In septic cardiomyocytes, mitochondria exhibit structural abnormalities, including swelling, cristae disruption, and altered membrane potential, accompanied by impaired oxidative phosphorylation and reduced ATP production. These alterations collectively compromise myocardial contractility and limit the energetic reserve required to sustain cardiac output under systemic inflammatory stress.

NF-κB contributes to mitochondrial dysfunction through multiple, interconnected mechanisms. Inflammatory mediators induced downstream of NF-κB signaling disrupt mitochondrial dynamics by altering the balance between mitochondrial fission and fusion, thereby impairing mitochondrial network integrity (96–100). In parallel, NF-κB-driven inflammatory signaling interferes with electron transport chain function, leading to inefficient oxidative phosphorylation and enhanced leakage of electrons that further increase mitochondrial reactive oxygen species (ROS) generation. This creates a vicious cycle in which mitochondrial dysfunction and inflammatory signaling mutually reinforce one another. Beyond these indirect effects, NF-κB also reshapes mitochondrial function at the transcriptional level. NF-κB-dependent gene expression programs influence key regulators of mitochondrial biogenesis, quality control, and stress adaptation, thereby modulating mitochondrial turnover and functional capacity during sepsis. Dysregulation of these adaptive responses limits the ability of cardiomyocytes to replace damaged mitochondria or restore bioenergetic efficiency, exacerbating metabolic insufficiency under sustained inflammatory stress. The resulting energy deficit has profound functional consequences for the heart. Reduced ATP availability disrupts calcium homeostasis and excitation–contraction coupling, impairing sarcoplasmic reticulum calcium cycling and diminishing contractile force generation. Importantly, these bioenergetic defects can occur even in the absence of irreversible structural myocardial damage, providing a mechanistic explanation for the characteristic reversible yet clinically significant cardiac depression observed in sepsis-induced myocardial injury (101) (**Figure 5**).

**Figure 5:**
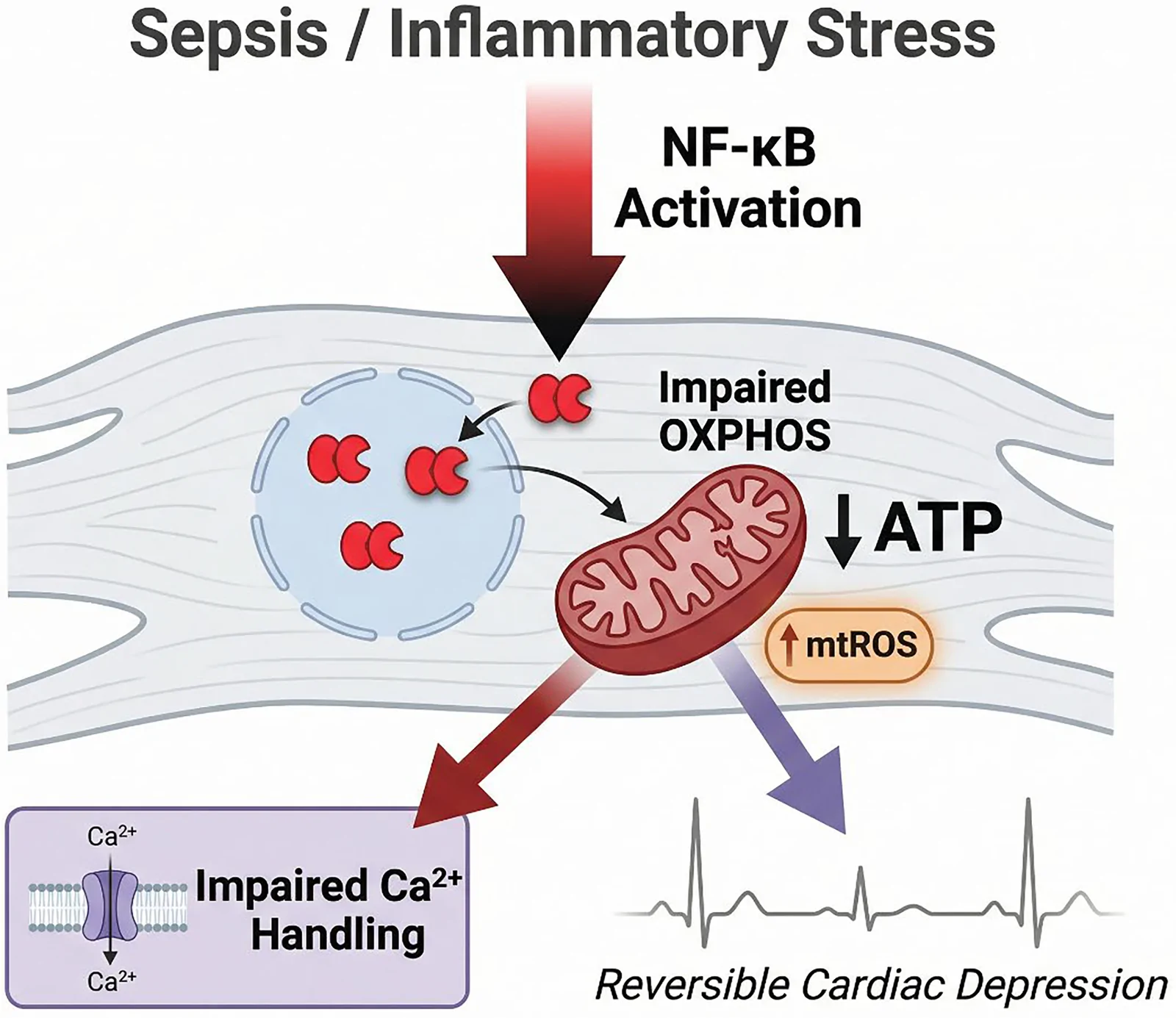
NF-κB-mediated mitochondrial dysfunction in septic myocardium. During sepsis, sustained NF-κB activation impairs mitochondrial function in cardiomyocytes, leading to defective oxidative phosphorylation and reduced ATP production. The resulting energy deficit disrupts calcium handling and excitation–contraction coupling, causing reversible myocardial depression despite the absence of overt structural damage.

Collectively, NF-κB-mediated mitochondrial dysfunction represents a critical mechanism by which inflammatory stress is translated into metabolic failure and functional impairment in the septic heart. By linking inflammatory signaling to impaired energy production, NF-κB sets the stage for cardiomyocyte vulnerability to downstream cell fate decisions, including apoptosis and other forms of regulated cell death discussed in subsequent sections.

### 4.4 Emerging links between NF-κB and ferroptosis-related metabolic stress

Beyond oxidative stress and mitochondrial dysfunction, ferroptosis has emerged as a metabolism-related form of regulated cell death potentially involved in sepsis-induced myocardial injury (91, 102). Compared with apoptosis and pyroptosis, however, the relationship between NF-κB signaling and ferroptosis in the septic myocardium remains less well established. Existing studies consistently show that septic myocardial injury is associated with iron overload, glutathione depletion, lipid peroxidation, mitochondrial damage, and altered expression of ferroptosis-related regulators such as GPX4, ACSL4, SLC7A11, and ferritinophagy-related molecules (103–105). Nevertheless, many of these studies primarily demonstrate the presence of ferroptosis during sepsis rather than directly proving that NF-κB is an upstream driver of ferroptotic cardiomyocyte death. In contrast, other studies suggest that inflammatory signaling, including NF-κB-related pathways, may indirectly enhance ferroptosis by increasing ROS production, disrupting redox homeostasis, and promoting pro-inflammatory cytokine release (106, 107). These findings indicate that NF-κB may contribute to a permissive inflammatory–oxidative environment for ferroptosis, but the direct molecular connection between NF-κB activation and ferroptosis execution in cardiomyocytes remains incompletely defined. This distinction represents an important knowledge gap. At present, it remains unclear whether NF-κB directly regulates ferroptosis-related genes in septic cardiomyocytes, whether its effects are mediated mainly through macrophage-derived inflammatory mediators, or whether ferroptosis-associated lipid peroxidation secondarily amplifies NF-κB signaling. Future studies should therefore combine cardiomyocyte-specific NF-κB manipulation with ferroptosis-specific interventions and *in vivo* septic myocardial injury models to clarify causality. Such studies will be essential for determining whether targeting the NF-κB–ferroptosis interface can simultaneously reduce inflammation, metabolic dysfunction, and cardiomyocyte loss in sepsis.

NF-κB may influence ferroptosis-related metabolic stress through regulation of iron metabolism, lipid peroxidation pathways, and inflammatory mediators that exacerbate redox imbalance. Conversely, lipid peroxidation products generated during ferroptosis can further activate inflammatory signaling, potentially feeding back into NF-κB activation. This bidirectional interaction positions ferroptosis as both a downstream effector and an amplifier of NF-κB-driven inflammatory stress (108–110). Although the precise mechanisms linking NF-κB to ferroptosis in septic myocardium remain incompletely defined, this emerging connection highlights a promising area for future research. Elucidating how NF-κB coordinates inflammatory signaling with ferroptosis-related metabolic stress may uncover novel therapeutic targets capable of simultaneously mitigating inflammation, metabolic dysfunction, and cardiomyocyte injury in sepsis (**Figure 6**).

**Figure 6:**
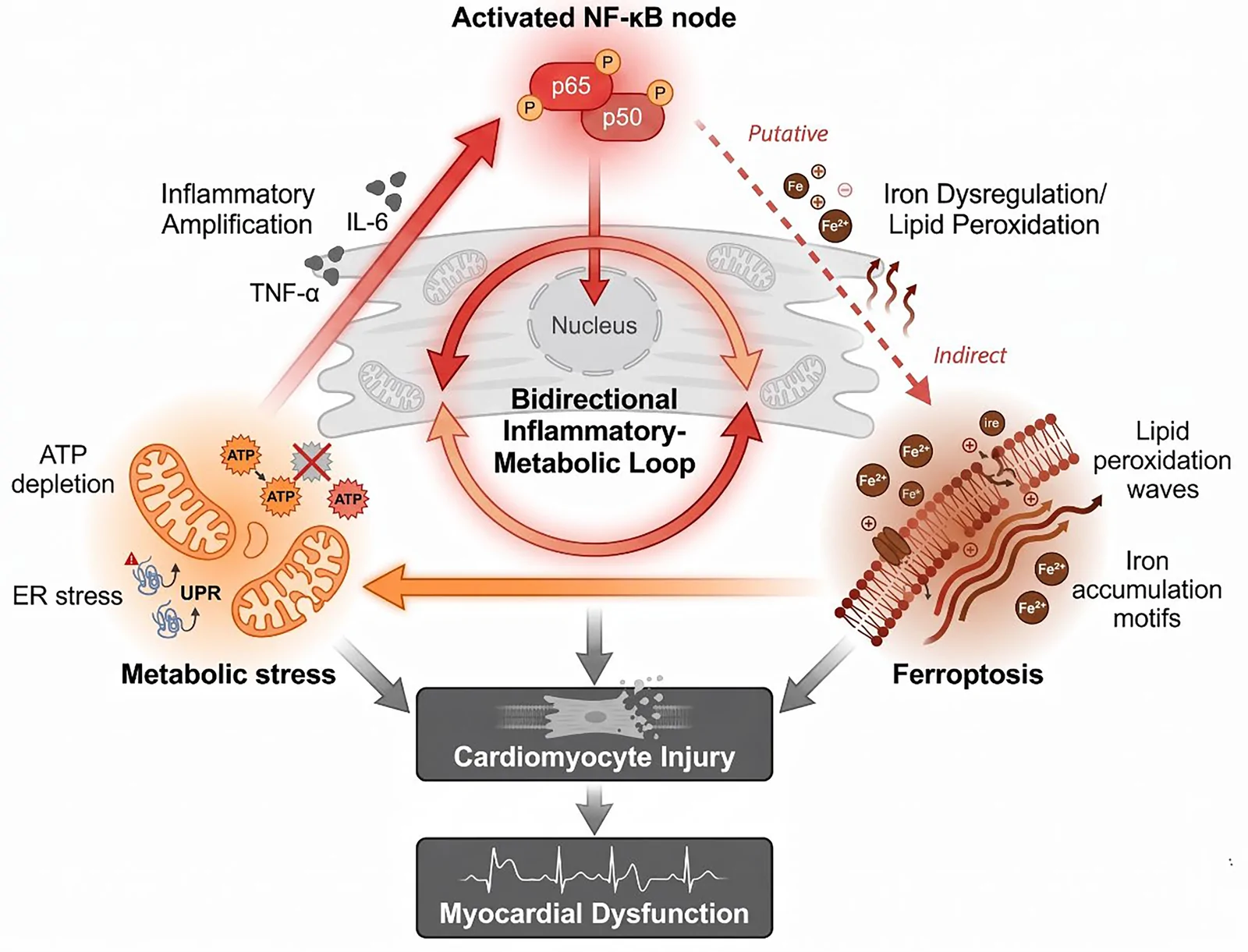
Emerging links between NF-κB signaling and ferroptosis-related metabolic stress in septic myocardium. NF-κB signaling may promote ferroptosis-related metabolic stress by regulating iron homeostasis, lipid peroxidation, and inflammatory mediators. Conversely, lipid peroxidation during ferroptosis can further amplify inflammatory signaling, forming a bidirectional inflammatory–metabolic loop that contributes to cardiomyocyte injury and myocardial dysfunction in sepsis.

## 5 NF-κB as a determinant of cardiomyocyte fate

Beyond orchestrating inflammatory and metabolic responses, NF-κB plays a decisive role in shaping cardiomyocyte fate during sepsis-induced myocardial injury. Cardiomyocytes exposed to septic stress face multiple, often competing, cell fate programs, including survival, apoptosis, pyroptosis, and ferroptosis. NF-κB signaling occupies a central position within this decision-making landscape, integrating inflammatory cues and metabolic stress signals to determine whether cardiomyocytes adapt, malfunction, or undergo regulated cell death.

### 5.1 Apoptosis: NF-κB-dependent survival–death balance

Apoptosis represents one of the most extensively studied forms of cardiomyocyte death in sepsis-induced myocardial injury. Inflammatory mediators released during sepsis activate intrinsic and extrinsic apoptotic pathways, culminating in caspase-3 activation and irreversible cellular dismantling (111). NF-κB exerts a context-dependent influence on apoptotic signaling. Under certain conditions, transient NF-κB activation can induce the expression of anti-apoptotic genes, promoting short-term cell survival and stress adaptation (112–116). However, sustained or excessive NF-κB signaling in the septic milieu shifts this balance toward apoptosis by amplifying inflammatory cytokine production, disrupting mitochondrial integrity, and enhancing pro-apoptotic signaling cascades. In cardiomyocytes, this prolonged inflammatory pressure overrides protective mechanisms, leading to caspase-3–mediated apoptosis and loss of functional myocardial mass. Thus, NF-κB does not uniformly suppress or promote apoptosis; rather, it modulates the survival–death equilibrium of cardiomyocytes in a manner dictated by the intensity and duration of septic stress.

### 5.2 Pyroptosis and inflammasome-associated injury

In addition to apoptosis, pyroptosis has emerged as a critical contributor to myocardial injury in sepsis. Pyroptosis is a highly inflammatory form of regulated cell death characterized by inflammasome activation, caspase-dependent membrane pore formation, and rapid release of pro-inflammatory cytokines (117–119). Unlike apoptosis, which is largely immunologically silent, pyroptosis actively amplifies inflammatory signaling and tissue injury, making it particularly relevant in the context of sepsis-induced myocardial dysfunction.

NF-κB signaling is indispensable for the priming phase of inflammasome activation, especially for the nucleotide-binding oligomerization domain-like receptor family pyrin domain-containing 3 (NLRP3) inflammasome. Through transcriptional upregulation of inflammasome components, including NLRP3 itself, as well as pro–interleukin-1β and pro–interleukin-18, NF-κB establishes a permissive intracellular environment that sensitizes cardiomyocytes and cardiac-resident immune cells to subsequent inflammasome activation (120). This priming step represents a critical checkpoint at which inflammatory signaling is translated into pyroptotic susceptibility. Once activated, inflammasome signaling initiates pyroptotic cell death and promotes the maturation and release of inflammatory cytokines, further exacerbating myocardial inflammation and dysfunction. In the septic heart, this process is not restricted to immune cells; accumulating evidence indicates that cardiomyocytes themselves can undergo inflammasome activation and pyroptosis under severe inflammatory and metabolic stress. Pyroptotic cardiomyocyte death directly reduces functional myocardial mass and disrupts cardiac contractility. Beyond direct cell loss, pyroptosis profoundly reshapes the myocardial inflammatory microenvironment. The release of damage-associated molecular patterns and cytokines from pyroptotic cells amplifies local immune activation and reinforces NF-κB signaling in surrounding cardiomyocytes and infiltrating immune cells. This establishes a self-perpetuating inflammatory loop, in which NF-κB-driven inflammation primes inflammasome activation, and inflammasome-mediated pyroptosis further intensifies NF-κB signaling and tissue injury (**Figure 7**).

**Figure 7:**
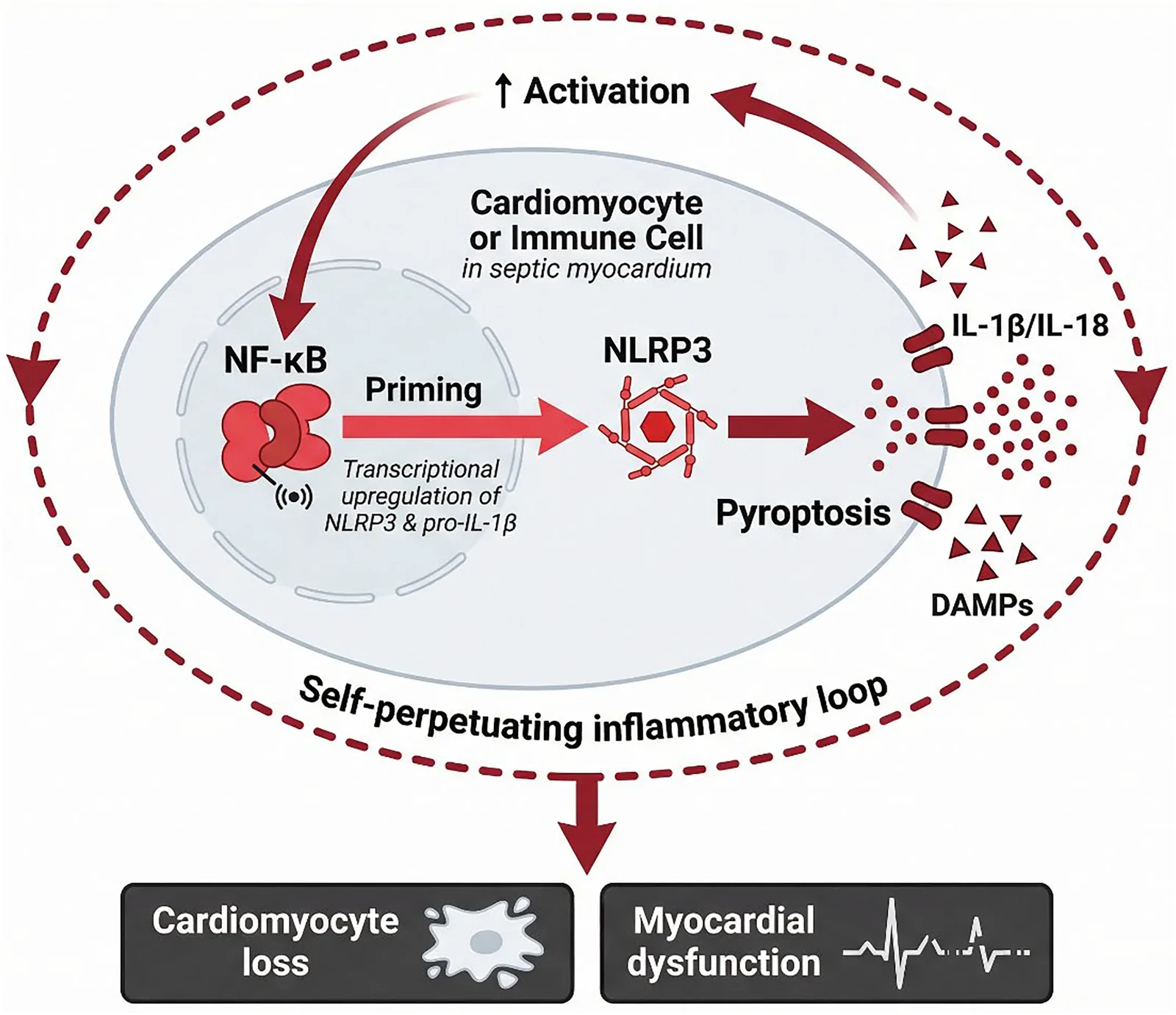
NF-κB–primed inflammasome activation and pyroptosis in septic myocardium. In sepsis, NF-κB signaling primes inflammasome activation by upregulating inflammasome components and pro-inflammatory cytokines. Subsequent inflammasome activation triggers pyroptosis, leading to cytokine and DAMP release that amplifies NF-κB signaling. This self-perpetuating inflammatory loop promotes cardiomyocyte loss and myocardial dysfunction.

Collectively, these mechanisms position pyroptosis as a pivotal link between inflammatory amplification and cardiomyocyte fate in sepsis-induced myocardial injury. Through its central role in inflammasome priming, NF-κB integrates inflammatory stress with pyroptotic cell death, thereby contributing to both myocardial dysfunction and sustained cardiac inflammation.

### 5.3 Ferroptosis and other non-apoptotic death modalities

Ferroptosis is increasingly recognized as a potential contributor to cardiomyocyte injury during sepsis, particularly because septic stress is characterized by oxidative injury, disturbed iron homeostasis, mitochondrial dysfunction, and lipid peroxidation (110, 121, 122). Compared with apoptosis and pyroptosis, however, ferroptosis should currently be viewed as a less established NF-κB-linked cell death mechanism in sepsis-induced myocardial injury. Existing studies support the presence of ferroptosis-related changes in septic myocardium, including altered GPX4, ACSL4, SLC7A11, FTH1, and NCOA4-related pathways, but direct evidence that NF-κB acts as an upstream transcriptional regulator of these ferroptosis-executing programs in cardiomyocytes is limited (123, 124).

Accordingly, the role of NF-κB in ferroptotic cardiomyocyte death should be interpreted with caution. Rather than directly executing ferroptosis, NF-κB may indirectly increase ferroptotic susceptibility by sustaining inflammatory cytokine production, increasing ROS generation, weakening antioxidant defenses, and promoting an oxidative microenvironment (125, 126). This indirect model is consistent with the broader concept that inflammation and lipid peroxidation can reinforce one another, but it remains unclear whether NF-κB directly binds and regulates core ferroptosis-related genes in septic cardiomyocytes. Therefore, the NF-κB–ferroptosis axis represents an important hypothesis-generating area for future research rather than a fully established mechanism. Clarifying this relationship will require cardiomyocyte-specific NF-κB knockout or inhibition models, ferroptosis-specific rescue experiments, lipid peroxidation measurements, and chromatin-based evidence such as ChIP-qPCR or ChIP-seq.

Taken together, these observations underscore a central concept: NF-κB is neither inherently pro-survival nor strictly pro-death in cardiomyocytes. Instead, NF-κB functions as a context-dependent determinant of cardiomyocyte fate, integrating inflammatory intensity, metabolic stress, and intracellular damage signals to bias the cell toward adaptation, dysfunction, or death. In sepsis-induced myocardial injury, this integrative role of NF-κB helps explain the coexistence of reversible myocardial depression and irreversible cardiomyocyte loss within the same pathological framework.

## 6 Functionally validated non-coding RNA and epigenetic regulation of NF-κB signaling

Non-coding RNAs add an important regulatory layer to NF-κB signaling in sepsis-induced myocardial injury, but the mechanistic evidence is highly uneven. Rather than cataloguing individual miRNAs, lncRNAs, and circRNAs, this section focuses on a small number of ncRNA–NF-κB axes supported by gain- or loss-of-function experiments and, where available, direct target validation. These examples illustrate three major regulatory modes: suppression of upstream NF-κB signaling, ceRNA-mediated control of NF-κB activators, and context-dependent modulation of inflammatory and cell-death programs.

### 6.1 Functionally validated ncRNA–NF-κB axes in sepsis-induced myocardial injury

MicroRNAs (miRNAs) represent a major class of post-transcriptional regulators that critically modulate NF-κB signaling by targeting key upstream adaptors, signaling intermediates, and regulatory nodes (127). Rather than acting as binary on–off switches, miRNAs function as signal rheostats, dynamically adjusting the intensity, timing, and persistence of NF-κB activation under septic conditions. A growing number of miRNAs have been shown to directly target adaptor molecules and signaling intermediates within the NF-κB pathway. By regulating proteins such as MyD88 and tumor necrosis factor receptor–associated factor 6 (TRAF6), miRNAs fine-tune signal transmission from innate immune receptors to the NF-κB transcriptional machinery, thereby controlling inflammatory amplification at an early and highly influential stage (128, 129). Through this mechanism, miRNAs can dampen excessive NF-κB activation without completely abolishing essential immune signaling. In addition to targeting positive regulators of NF-κB signaling, certain miRNAs modulate negative feedback mechanisms within inflammatory networks. For example, miRNA-mediated regulation of dual-specificity phosphatase 1 (DUSP1) indirectly shapes NF-κB activity through crosstalk with MAPK pathways, influencing both the magnitude and duration of inflammatory responses (130). This layered regulation underscores the ability of miRNAs to coordinate NF-κB and MAPK signaling dynamics rather than acting on isolated pathways. In the septic myocardium, dysregulation of miRNA expression disrupts these finely balanced control mechanisms, resulting in sustained NF-κB activation, excessive inflammatory stress, and subsequent metabolic and functional impairment. Aberrant miRNA profiles contribute to persistent cytokine production, oxidative stress, and mitochondrial dysfunction, thereby exacerbating cardiomyocyte injury. Conversely, restoration of miRNA balance has been shown to attenuate NF-κB-driven inflammation, normalize stress signaling, and protect against myocardial dysfunction (**Figure 8**).

**Figure 8:**
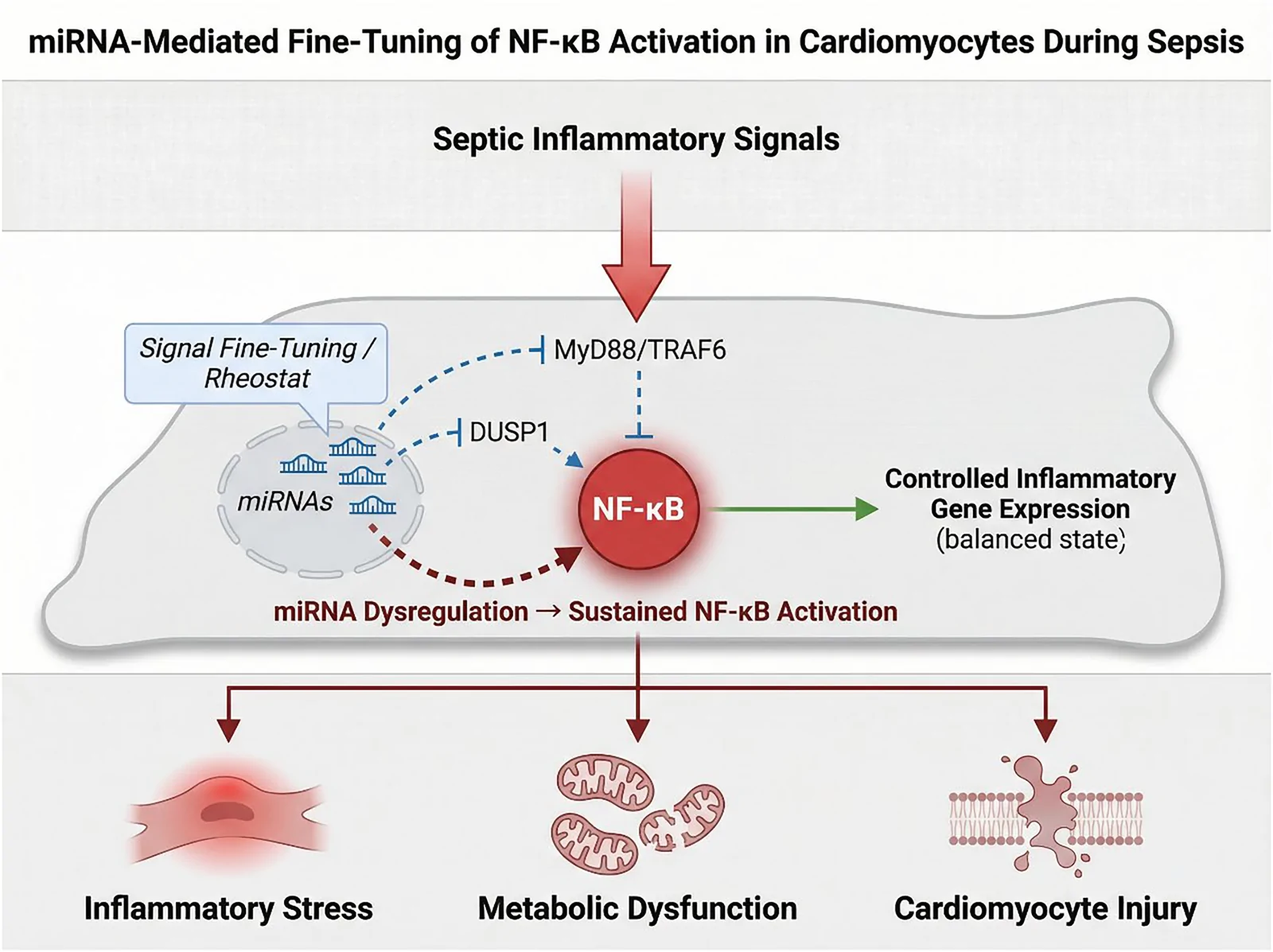
miRNA-mediated fine-tuning of NF-κB signaling in septic myocardium. MicroRNAs act as post-transcriptional rheostats that modulate NF-κB signaling by targeting upstream adaptors and regulatory nodes. By adjusting the intensity and duration of NF-κB activation, miRNAs prevent excessive inflammatory amplification while preserving essential immune responses. Dysregulation of this control contributes to sustained inflammation, metabolic stress, and cardiomyocyte injury in sepsis.

Collectively, these findings highlight miRNAs as critical modulators that confer precision and flexibility to NF-κB signaling in sepsis-induced myocardial injury. By fine-tuning signal amplitude and duration, miRNAs transform NF-κB from a rigid inflammatory pathway into a context-sensitive regulatory network, reinforcing their importance as upstream determinants of inflammatory, metabolic, and cardiomyocyte fate outcomes rather than mere downstream effectors.

### 6.2 Mechanistically defined lncRNA–NF-κB axes: MIAT and GAS5

MIAT provides one of the clearest examples of a mechanistically defined lncRNA–NF-κB axis in septic myocardial injury. In LPS-induced septic mice and HL-1 cardiomyocytes, MIAT expression was increased, whereas MIAT knockdown attenuated inflammatory responses and oxidative stress (129). Mechanistically, MIAT directly interacted with miR-330-5p, thereby relieving miR-330-5p-mediated repression of TRAF6 and promoting downstream NF-κB activation. Dual-luciferase assays validated both the MIAT–miR-330-5p and miR-330-5p–TRAF6 interactions, providing direct evidence for a MIAT/miR-330-5p/TRAF6/NF-κB ceRNA axis. Consistently, an independent study reported that MIAT silencing reduced NF-κB-associated inflammation, oxidative stress, apoptosis, and myocardial dysfunction in experimental sepsis. Thus, MIAT illustrates how a lncRNA can amplify NF-κB signaling through a defined post-transcriptional regulatory circuit rather than through a broadly inferred scaffold function (**Figure 9**).

**Figure 9:**
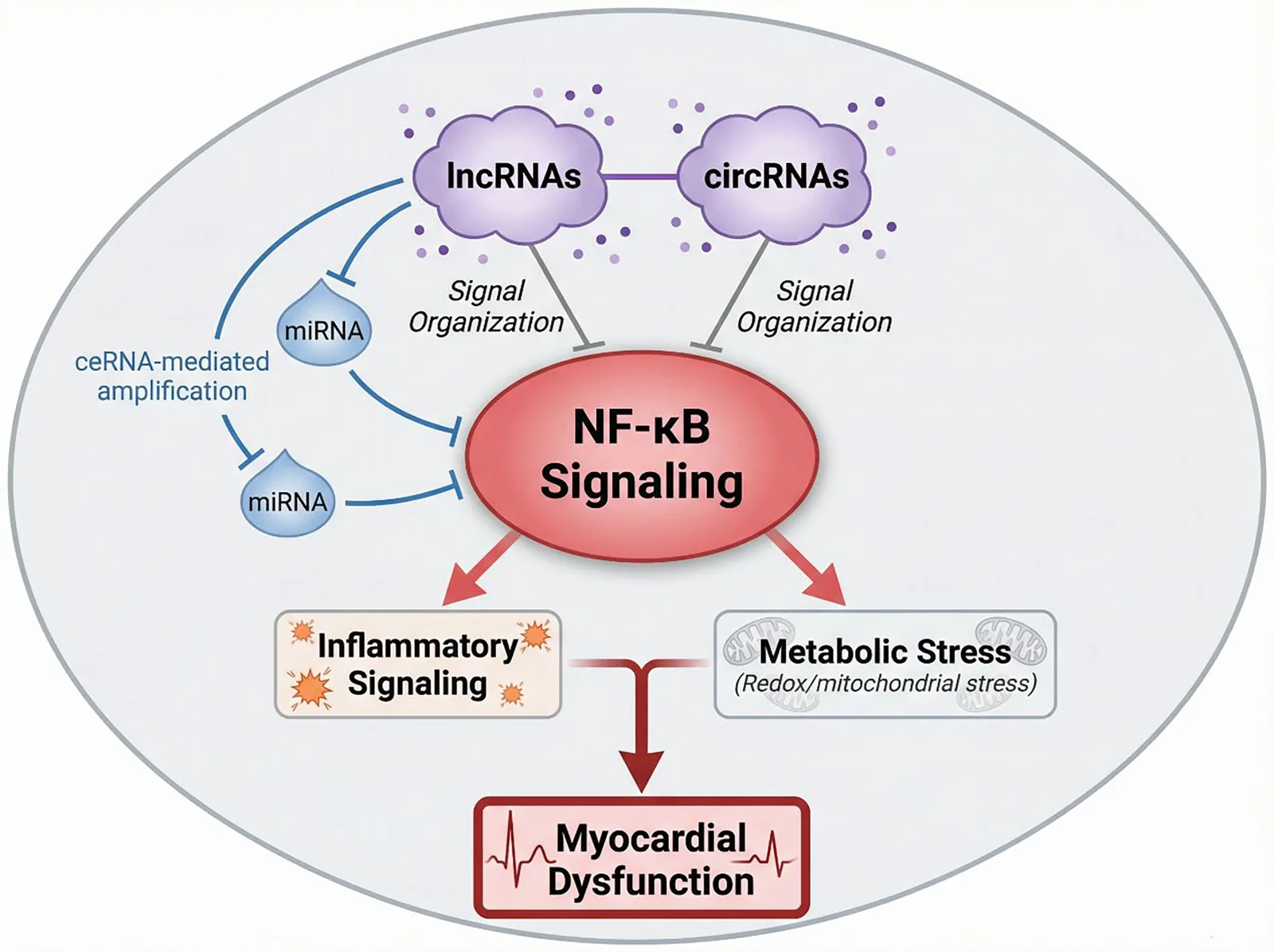
lncRNA- and circRNA-mediated regulation of NF-κB signaling in septic myocardium. Long non-coding RNAs and circular RNAs act as higher-order regulators of NF-κB signaling by functioning as molecular scaffolds and competing endogenous RNAs. Through organizing signaling complexes and sequestering miRNAs, these non-coding RNAs amplify and sustain NF-κB activity, linking inflammatory signaling to metabolic stress and myocardial dysfunction in sepsis.

### 6.3 Isoform-specific epigenetic regulation of NF-κB: the example of HDAC9

Epigenetic regulation of NF-κB signaling should be considered at the level of individual HDAC isoforms rather than HDACs as a uniform enzyme family. Class I HDACs, including HDAC1, HDAC2, and HDAC3, are predominantly associated with nuclear transcriptional regulation, whereas class II HDACs show more diverse subcellular localization and non-histone substrates (131, 132). Importantly, these isoforms may exert distinct, and in some settings opposing, effects on cardiac inflammatory signaling. In cardiac myocytes, HDAC1 has been shown to repress TNF-α-induced NF-κB transcriptional activity, indicating that loss of HDAC1 activity does not necessarily produce a uniformly anti-inflammatory or cardioprotective effect (133). In contrast, HDAC3 facilitates inflammatory signaling in LPS-treated cardiomyocytes, where LPS-induced mitochondrial ROS/c-Src-dependent HDAC3 activation promotes TNF-α expression and NF-κB/p65 accumulation at the TNF-α promoter (134). These observations illustrate the functional heterogeneity even within Class I HDACs.

Class II HDACs add another layer of complexity. HDAC6 is a Class IIb HDAC that is predominantly cytoplasmic and regulates the acetylation of multiple non-histone proteins (135, 136). Selective HDAC6 inhibition suppresses oxidative stress, TLR4–MAPK/NF-κB activation, and pro-inflammatory cytokine production in LPS-stimulated macrophages, although direct evidence in septic cardiomyocytes remains limited (137). By contrast, HDAC9 has been directly implicated in experimental sepsis-induced myocardial injury. HDAC9 is upregulated in LPS-treated rat myocardium and H9C2 cells, whereas HDAC9 knockdown reduces p-IκBα and p-p65, inhibits p65 nuclear translocation, decreases inflammatory cytokine production and apoptosis, and improves cardiac function (138). Thus, among the HDAC isoforms discussed here, HDAC9 currently has relatively direct functional evidence linking it to NF-κB-associated myocardial injury during sepsis.

These isoform-specific effects have important therapeutic implications. Broad-spectrum HDAC inhibition may simultaneously suppress detrimental HDAC activities while interfering with isoforms that support adaptive transcriptional or cardiac responses. Moreover, pan-HDAC inhibitors have been associated with systemic toxicities, including thrombocytopenia, fatigue, gastrointestinal adverse effects, and, for some agents, cardiac toxicity (139–141). Therefore, therapeutic development in sepsis-induced myocardial injury should prioritize isoform-selective modulation and define the relevant cell type, disease stage, and downstream substrate rather than assuming that global HDAC inhibition will be uniformly beneficial (**Figure 10**).

**Figure 10:**
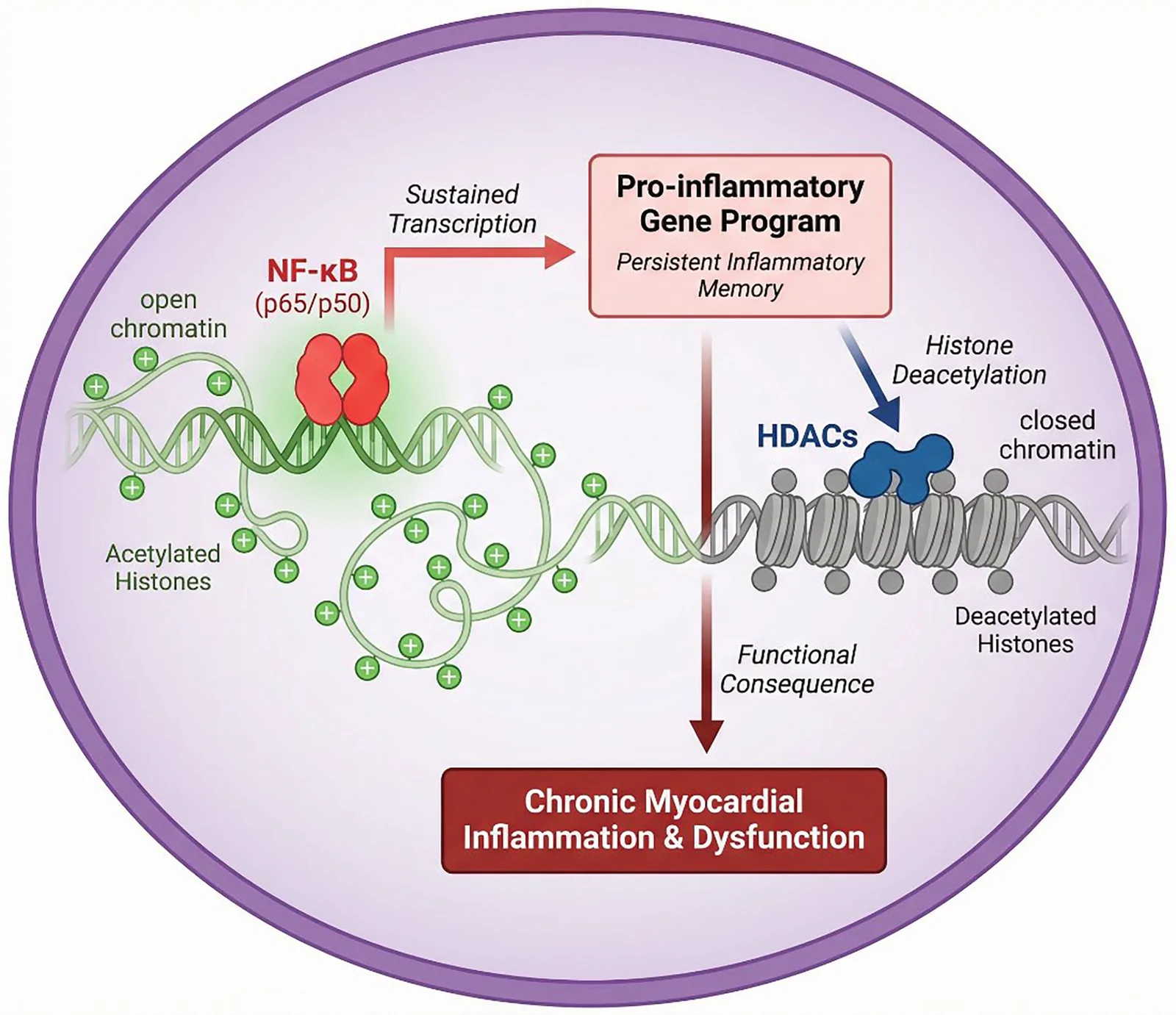
Epigenetic regulation of NF-κB transcriptional output in septic myocardium. Epigenetic modulators, particularly histone deacetylases, shape NF-κB–dependent transcription by controlling chromatin accessibility at inflammatory gene loci. Dysregulated epigenetic regulation skews NF-κB transcription toward sustained pro-inflammatory programs, contributing to persistent myocardial inflammation and dysfunction during sepsis.

Collectively, non-coding RNAs and epigenetic mechanisms add critical layers of regulation to NF-κB signaling, enabling precise control over inflammatory intensity, metabolic stress responses, and cell fate decisions. By fine-tuning signal amplitude and transcriptional specificity, these regulatory systems transform NF-κB from a linear inflammatory pathway into a highly adaptable signaling hub capable of orchestrating complex myocardial responses during sepsis. Although several circRNAs have also been linked to inflammatory signaling in sepsis, direct mechanistic evidence connecting specific circRNAs to NF-κB regulation in cardiomyocytes remains comparatively limited; therefore, they are not discussed in detail here.

## 7 Therapeutic implications: targeting NF-κB-centered networks

The recognition of NF-κB as a central integrator of inflammation, metabolic dysfunction, and cardiomyocyte fate has important therapeutic implications for sepsis-induced myocardial injury. However, NF-κB-related interventions should not be regarded as a single therapeutic category. A critical distinction must be made between direct NF-κB pathway modulators and agents that only indirectly or secondarily reduce NF-κB activity. Direct NF-κB-targeted strategies include interventions that inhibit IKK activation, prevent IκBα degradation, block p65 phosphorylation or nuclear translocation, or interfere with NF-κB-dependent transcription. In contrast, many pharmacological agents, natural compounds, antioxidants, RAAS modulators, and ncRNA-based approaches reduce NF-κB activity as part of broader anti-inflammatory, antioxidant, metabolic, or immunomodulatory effects. These agents should therefore be described as NF-κB-modulating or NF-κB-associated interventions rather than pathway-specific NF-κB-targeted therapies. This distinction is important because therapeutic specificity determines both mechanistic interpretation and translational feasibility.

### 7.1 Protective versus detrimental roles of NF-κB

NF-κB signaling exhibits a dual role in sepsis-induced myocardial injury, with context-dependent protective versus detrimental effects (111, 142). Transient or moderate NF-κB activation in cardiomyocytes can induce protective gene programs, including upregulation of anti-apoptotic factors such as Bcl-2 and Bcl-xL, and contributes to ischemic or stress preconditioning-mediated cardioprotection (143). Additionally, A20 (TNFAIP3), an NF-κB target and negative feedback regulator, mitigates excessive inflammatory responses and limits myocardial damage (144). In contrast, sustained or unrestrained NF-κB activation amplifies pro-inflammatory cascades, metabolic dysfunction, and cell death pathways, contributing to organ injury in sepsis and associated immune paralysis (145). Moreover, broad or systemic inhibition of NF-κB has been shown to impair host defense mechanisms and increase susceptibility to secondary infections, highlighting the risk of indiscriminate pathway suppression. These observations underscore the importance of context- and tissue-specific modulation strategies that balance NF-κB’s protective functions while limiting its detrimental impact.

### 7.2 Pharmacological modulation of NF-κB signaling

Pharmacological agents that indirectly or directly modulate NF-κB signaling have demonstrated protective effects against septic myocardial injury in preclinical studies. Interventions targeting the renin–angiotensin–aldosterone system (RAAS) can attenuate NF-κB activation by suppressing upstream inflammatory signaling and oxidative stress, thereby reducing myocardial inflammation and dysfunction (146–150). Anesthetic and sedative agents used in the intensive care unit have also been shown to modulate NF-κB-driven inflammatory responses, mitigating myocardial injury via immunomodulatory and neuroimmune mechanisms. Antioxidant strategies can reduce ROS accumulation and restore redox balance, indirectly dampening NF-κB activation and disrupting the feed-forward loop between inflammation and oxidative stress.

#### 7.2.1 Clinical relevance and limitations

Despite promising preclinical results, direct translation to patients is constrained by heterogeneity in septic populations, the timing of intervention relative to disease progression, and potential effects on host defense mechanisms. The optimal dose, duration, and patient selection criteria remain to be determined.

### 7.3 Natural compounds and multi-target interventions

Natural compounds and plant-derived bioactive molecules have emerged as promising modulators of NF-κB-centered networks in sepsis-induced myocardial injury. Unlike single-target synthetic agents, many natural compounds exert multi-target effects, simultaneously suppressing NF-κB signaling, attenuating oxidative stress, and modulating metabolic and cell death pathways (151, 152). This pleiotropic nature may be particularly advantageous in the complex and multifactorial context of sepsis. Experimental studies have demonstrated that various natural compounds can reduce myocardial inflammation, improve mitochondrial function, and limit cardiomyocyte injury through coordinated inhibition of NF-κB and related signaling pathways. These findings support the concept that multi-target interventions may more effectively counteract the interconnected pathological processes underlying septic myocardial dysfunction. Nevertheless, several limitations hinder the clinical translation of natural compounds. Variability in compound composition, limited bioavailability, uncertainty regarding optimal dosing, and potential off-target effects present significant challenges. Moreover, the lack of standardized formulations and rigorous clinical trials restricts their immediate applicability. Addressing these limitations will be essential for harnessing the therapeutic potential of natural compounds in targeting NF-κB-centered networks. Clinical relevance and limitations: Most evidence derives from *in vitro* or animal models, and human safety, pharmacokinetics, and effective dosing require further investigation before clinical application.

### 7.4 ncRNA-based and upstream pathway-targeted strategies

Advances in molecular biology have opened new avenues for targeting NF-κB signaling with greater precision. Non-coding RNA–based strategies, including miRNA mimics, inhibitors, and lncRNA or circRNA modulators, offer the possibility of selectively fine-tuning NF-κB activation at critical regulatory nodes. By targeting upstream adaptors such as MyD88 or TRAF6, or modulating negative regulators of inflammatory signaling, ncRNA-based interventions may achieve more nuanced control over NF-κB signaling than conventional pharmacological inhibitors (106). Targeting upstream pattern recognition receptors and signaling adaptors also represents an attractive strategy for interrupting NF-κB activation at its source. Such approaches may prevent inflammatory amplification before it propagates into metabolic dysfunction and cardiomyocyte injury. However, given the central role of NF-κB in host defense, excessive or non-selective inhibition carries the risk of immunosuppression and impaired pathogen clearance. A major challenge for ncRNA-based and upstream-targeted therapies lies in delivery and safety. Achieving efficient, tissue-specific delivery to the myocardium while minimizing systemic effects remains a significant hurdle. Additionally, long-term safety, off-target effects, and immune activation by RNA-based therapeutics require careful evaluation before clinical application.

Collectively, these therapeutic strategies underscore the translational relevance of NF-κB-centered signaling networks in sepsis-induced myocardial injury. Rather than advocating indiscriminate suppression of NF-κB, emerging evidence supports a more refined approach aimed at context-dependent modulation of NF-κB activity. By targeting key nodes within inflammatory, metabolic, and cell fate networks, it may be possible to preserve essential immune functions while mitigating myocardial injury. Current therapeutic strategies targeting NF-κB-centered regulatory networks in sepsis-induced myocardial injury are summarized in **Table 4**.

**Table 4:** Therapeutic strategies targeting NF-κB-centered regulatory networks.

Strategy category	Representative approaches	NF-κB-related targets	Translational considerations
Pharmacological modulation	RAAS inhibitors, sedatives	TLR4–NF-κB, NF-κB/STAT3	Clinically available, timing-dependent
Antioxidant strategies	ROS scavengers, NRF2 activation	NF-κB–ROS feedback loop	Limited efficacy as monotherapy
Natural compounds	Multi-component phytochemicals	NF-κB, MAPK, oxidative stress	Multi-target benefit, standardization needed
ncRNA-based therapies	miRNA mimics/inhibitors	MyD88, TRAF6, DUSP1	High precision, delivery challenges
Upstream targeting	TLR4 or adaptor modulation	NF-κB activation source	Risk of immunosuppression

### 7.5 Integrative considerations for clinical translation

Although preclinical studies support several strategies for modulating NF-κB-centered networks, their clinical translation remains constrained by patient heterogeneity, disease-stage dependence, and the essential role of NF-κB in antimicrobial defense. These considerations indicate that therapeutic application will require biomarker-guided patient selection and precise timing rather than uniform pathway inhibition.

### 7.6 Biomarker-guided stratification and the preclinical-to-clinical gap

A major translational challenge is that NF-κB modulation is unlikely to benefit all patients with sepsis-induced myocardial injury equally. Because NF-κB contributes not only to maladaptive inflammation but also to antimicrobial defense and cell survival, therapeutic responses may depend strongly on the inflammatory state, disease stage, and extent of myocardial involvement. A clinically useful stratification strategy would therefore likely require a combination of pathway-proximal, systemic inflammatory, and cardiac injury biomarkers rather than reliance on a single marker. Pathway-proximal measures such as phosphorylated p65 are mechanistically attractive indicators of NF-κB activity, but their clinical application remains largely investigational. Studies in patients with sepsis have demonstrated altered NF-κB activation and phosphorylation responses in circulating immune cells, although standardized assays and validated thresholds for therapeutic stratification are currently lacking (153, 154). More readily measurable circulating inflammatory biomarkers, particularly IL-6, may help characterize systemic inflammatory activity and have shown associations with disease severity and clinical outcomes in patients with sepsis (155–157). Cardiac-specific biomarkers such as cardiac troponin and BNP or NT-proBNP, together with echocardiographic evidence of ventricular dysfunction, may help identify concurrent myocardial injury or cardiac dysfunction (6, 158–160). Importantly, none of these biomarkers has yet been validated as a stand-alone companion marker for selecting patients for NF-κB-directed therapy. Future studies should therefore evaluate multimarker panels integrating inflammatory activity, myocardial injury, and cardiac function to determine whether specific biological phenotypes are more likely to respond to NF-κB-modulating interventions.

Patient selection should also consider the temporal evolution of sepsis. Individuals with persistent evidence of excessive inflammatory activation and concomitant myocardial dysfunction may represent an important population for future evaluation of NF-κB-modulating strategies. In contrast, patients showing features of immune suppression, such as persistent lymphopenia, recurrent secondary infection, or relatively low inflammatory responsiveness, may be less suitable for further NF-κB suppression and would require particularly cautious assessment. Human studies have demonstrated that sepsis can be accompanied by reduced NF-κB responsiveness in circulating immune-cell populations, emphasizing that NF-κB activity is dynamic rather than uniformly increased throughout the disease course (153, 154). Thus, the predictive value of biomarkers such as p65 phosphorylation, IL-6, troponin, and BNP should ideally be evaluated longitudinally rather than at a single time point, because both systemic inflammation and septic cardiac dysfunction can evolve substantially during disease progression (6, 156).

A further limitation is the substantial gap between experimental efficacy and clinical validation. Most mechanistic and therapeutic studies of NF-κB-centered interventions in sepsis rely on LPS-treated cells, endotoxemia models, or cecal ligation and puncture models. Although these systems are valuable for defining molecular mechanisms, they cannot fully reproduce the heterogeneity of human sepsis, including differences in infectious source, age, comorbidities, immune status, supportive treatment, and the timing and phenotype of organ dysfunction (161–164). More broadly, numerous interventions showing benefit in experimental sepsis have failed to demonstrate comparable efficacy in clinical trials, highlighting persistent limitations in patient selection, disease modeling, intervention timing, and translation of preclinical endpoints into clinically meaningful outcomes (165–167). This limitation is particularly relevant to sepsis-induced myocardial injury, because most pharmacological agents, natural compounds, and ncRNA-based strategies discussed in this review have not been specifically evaluated in adequately powered clinical trials enrolling septic patients with objectively defined cardiac dysfunction (6, 168, 169). Future translational studies should therefore incorporate prospectively defined cardiac phenotypes, serial biomarker measurements, standardized echocardiographic assessment, and clinically meaningful outcomes. Such an approach will be essential for determining whether NF-κB-centered interventions can move beyond mechanistic promise toward safe and effective patient-specific therapy.

## 8 Conclusions and future perspectives

Sepsis-induced myocardial injury represents a complex and multifactorial pathological process in which inflammatory stress, metabolic dysfunction, and cardiomyocyte fate are tightly interconnected. Accumulating evidence synthesized in this review supports the concept that NF-κB signaling functions as a central integrator at the crossroads of these processes, rather than as a simple linear inflammatory pathway. By coordinating immune activation, redox imbalance, mitochondrial dysfunction, and regulated cell death programs, NF-κB shapes both the severity and reversibility of myocardial injury during sepsis. Importantly, this integrative role of NF-κB helps reconcile seemingly paradoxical observations in septic cardiomyopathy, such as the coexistence of transient myocardial depression and irreversible cardiomyocyte loss. NF-κB signaling does not uniformly promote survival or death; instead, it operates as a context-dependent regulatory hub that interprets inflammatory intensity and metabolic stress to determine cardiomyocyte fate. This conceptual framework provides a more cohesive understanding of septic myocardial dysfunction and underscores the limitations of reductionist, single-pathway models.

Looking forward, several key directions are likely to advance the field. First, single-cell and spatial transcriptomic approaches hold great promise for dissecting the myocardial immune microenvironment in sepsis. These technologies will enable precise mapping of NF-κB activation states across cardiomyocytes, resident immune cells, and infiltrating leukocytes, thereby revealing cell-type-specific signaling programs and intercellular communication networks that are obscured in bulk analyses. Such insights are essential for understanding how NF-κB-driven inflammation and metabolic stress are spatially organized within the septic heart. Second, the development of cardiomyocyte-specific or tissue-targeted NF-κB modulation strategies represents a critical translational goal. Given the indispensable role of NF-κB in host defense, systemic inhibition is unlikely to be safe or effective. Future therapeutic approaches should aim to selectively modulate NF-κB activity within the myocardium or specific immune cell subsets, preserving protective immune responses while limiting cardiac injury. Advances in targeted delivery systems, including nanoparticle-based and RNA-guided platforms, may facilitate this level of precision. Third, emerging evidence strongly supports the need for combination strategies rather than single-point interventions. Because NF-κB integrates inflammatory, metabolic, and cell death pathways, targeting one downstream effector in isolation is unlikely to fully restore myocardial homeostasis. Rational combination approaches that simultaneously attenuate inflammatory amplification, correct metabolic stress, and stabilize cardiomyocyte fate may offer superior efficacy. Such strategies should be guided by mechanistic insights into NF-κB-centered network dynamics and tailored to the temporal stages of sepsis. In conclusion, framing NF-κB as a central integrator of inflammation, stress-induced metabolic dysfunction, and cardiomyocyte fate provides a unifying perspective on sepsis-induced myocardial injury. Continued integration of systems-level analyses with targeted therapeutic development will be essential for translating this conceptual advance into meaningful clinical benefit for patients with sepsis.
